# Supplementary material for: Lymph Node Reporting and Data System (LN-RADS)—Retrospective Evaluation for Ultrasound Classification of Superficial Lymph Nodes
Source: Cancers (Basel). 2025 Jun 18;17(12):2030. doi: 10.3390/cancers17122030 (PMC12191221; doi:10.3390/cancers17122030)
Supplement: Supplementary file 1 [file cancers-17-02030-s001.zip › cancers-3664618-supplementary.pdf]

Table S1. Subjective morfological features of LNs. Ddetaild comnety on each feature is given below.

Shape:<br>1 - very long and slim<br>2 - more long than oval<br>3 - oval - proportion 1/2<br>4 - more round than oval<br>5 – round	Cortex irregularity:<br>1 - perfect regular<br>2 - subtle irregularity<br>3 - moderate irregularity<br>4 - strong irregularity, also FCT<br>5 - total infiltration, no hilum	Cortex echogeneity:<br>1 - strong hiperechoic<br>2 - moderate hiperechoic<br>3 - izoechoic<br>4 - subtle hypoechoic<br>5 - strong hypoechoic
Cortex inhomogeneity<br>1 - perfect homogenous<br>2 - homogenous<br>3 - average subtle inhomogeneity (may be due to technical artifacts)<br>4 - moderate inhomogeneity<br>5 - evident inhomogeneity	The margines<br>1 - perfect distinct outline<br>2 - distinct outline<br>3 - subtle borders' blur (may be due to technical artifacts)<br>4 - moderate border’s blur<br>5 - evident blur (extracapsular infiltration)	Colour Doppler vascular pattern<br>1 - no visible blood flow<br>2 - hilar flow (small tree)<br>3 - hilar-cortical flow (big tree)<br>4 - peripheral *<br>5 - vascular chaos

* - group 4 includes LNs with peripheral-type vascularization, which can take two forms: the first, where blood flow is present exclusively in the peripheral zone of the node, and the second, where central vessels extend peripherally to the capsule. In both cases, we observed an increased number of malignant LNs.
